# Radical care: voetvroue and the reclamation of pleasure in reproductive health

**DOI:** 10.3389/fgwh.2025.1531915

**Published:** 2025-08-26

**Authors:** Tamia Bianca Botes

**Affiliations:** ^1^ Department of Anthropology, University of the Witwatersrand, Johannesburg, South Africa; ^2^ Department of Anthropology, Sol Plaatje University, Kimberley, South Africa

**Keywords:** obstetric violence, midwifery, radical care, apartheid archive, TBAs, Eldorado Park

## Abstract

Obstetric violence, rooted in the racialised and gendered logics of colonial medicine, has long served as a tool for disciplining reproductive bodies. In both 19th-century Antebellum slavery and the Cape colony, Black women's bodies became sites of medical experimentation, regulation, and control. Gynaecology emerged as a site of race-making, displacing Black autonomous midwives and erasing their knowledge from official medical archives. Yet this erasure was never complete. In Eldorado Park, Black autonomous midwives, or voetvroue, have cultivated grounded, place-based forms of reproductive care: treating infertility, facilitating births, and enacting rituals transmitted along familial and communal lines. Drawing on archival research and life history interviews, this paper traces the erasure of “voetvroue”, or Black autonomous midwives, from the medical archive and discusses the colonial transformation of birth and obstetrics into a site of surveillance, control, and violence. It follows the lives of three voetvroue—Aunty Faeeza, Aunt Rose, and their grandmother, Ouma—who re-fashioned her two-bedroom backroom in Eldorado Park into a birthing space, or “hospitaal”. I argue that the huis-hospitaal constitutes a radical commons of care that offers a counter-space to colonial biomedical logics not through overt refusal but through the everyday enactment of pleasure, dignity, and agency. Here, pleasure is conceptualised as emotional, spiritual, and relational: a mode of re-imagining reproductive justice beyond the confines of state-sanctioned care. By reframing reproductive health through the lens of radical care, voetvroue reclaim space, knowledge, and autonomy for Black birthing women in the face of ongoing racial-capitalist violence. In doing so, they revalorise locale-specific modes of knowledge and technologies and prioritise holistic approaches to birthing care.

## Introduction

This paper emerges from writing and knowing through an amputated archive—one that is partial, fragmented, and epistemically violent. In the context of South African reproductive health histories, the knowledge and contributions of Black autonomous midwives have been systematically excluded or rendered invisible. So much so that Deacon (1) asserts that, from 1865 onward, the Black autonomous midwife *disappears* from the public archive. My methodological approach, developed through my Master's research, responds to this epistemic rupture. Rather than attempting to “fill in” what is missing, it engages the historical silence by reading across absence—tracing institutional logics within the archive while listening for what those archives cannot contain.

My archival research critically maps the institutionalisation of reproductive health in South Africa through systems of control that were racialised, gendered, and colonial in logic. I focus, in particular, on the historical displacement of Black midwifery and the rise of Western obstetrics as a professionalised, white, male domain. This was not incidental displacement, but a violently orchestrated process. In both Antebellum America and the Cape Colony, Black women's bodies were subjected to experimental procedures and became key sites of medical surveillance. At the same time, Indigenous knowledge systems and care practices embodied by Black midwives were actively erased. These two processes—the disciplining of Black women's bodies and the erasure of their expertise—were mutually reinforcing. White male physicians and accoucheurs leveraged the authority of science to regulate and control reproduction, positioning gynaecology and obstetrics as legitimate while rendering indigenous and communal forms of knowledge as “backward” or dangerous (2). While I juxtapose Antebellum US and the Cape, I treat them as connected but distinct regimes; the comparison is analytic rather than an assertion of equivalence. In the South African context, as Deacon (1) showed, the disappearance of Black midwives from the public archive was not due to a lack of activity but rather a systematic process of erasure. The archive, therefore, does not merely reflect absence—it produces it. Reading the archive with suspicion, and alongside its omissions, allows for a reframing of obstetric violence as a structural feature of colonial and apartheid modernity, one that was enacted through bureaucracies of health, race, and gender.

While the term “obstetric violence” remains contested (3), I use it here to signal the systemic and often racialised abuse, coercion, and neglect experienced by Black women in medical birthing institutions, both historically and in the present. It includes verbal abuse, forced procedures, the disregard for consent, and the silencing of cultural and embodied knowledges during birth (3). Importantly, obstetric violence also extends to midwives themselves—particularly Black autonomous midwives—whose exclusion, surveillance, and epistemic devaluation constitute a form of institutional violence. Their knowledge systems have been dismissed, their authority undermined, and their practices either erased or co-opted by medical institutions, reinforcing the patriarchal and racialised hierarchies that govern reproductive care.

Countering the institutional archive, life history interviews function both as a method of historical recovery and a practice of epistemic reorientation. These interviews were conducted with 11 voetvroue in Eldorado Park. This paper, however, focuses on the lives of three: Aunty Faeeza, Aunt Rose, and their grandmother, Ouma. Spanning three generations, these women have trained under one another and are recognised as voetvroue by their community. Their narratives offer a textured account of birthing care as communal, relational, and spiritually grounded—carried through rituals, herbal knowledge, and intergenerational teaching. These stories do more than humanise the archive's silences; they assert alternative ways of knowing and doing that have survived despite historical and archival erasure. The huis-hospitaal they crafted was their home—a modest two-bedroom backroom in Eldorado Park, never intended as a birthing space but gradually became one. It exemplifies what I call a *radical commons of care*: an ordinary home transformed into a birthing space through everyday practices that centred dignity, agency, and well-being. The women centre what mainstream biomedicine often forecloses: *pleasure*.

As conceptualised in this paper, pleasure is not confined to bodily sensation or individual satisfaction, but it is understood as a relational, emotional, and spiritual experience that reclaims the sacredness of birth. It encompasses the right to dignity, choice, and holistic well-being in reproductive care. In the huis-hospitaal, pleasure is embedded in ritual, intimacy, and the restoration of control over one's own body and birthing process. This framing draws on Black feminist thought, particularly Audre Lorde's assertion that the erotic, understood expansively as embodied joy, feeling, and connection, is a profound source of political power and resistance (4). Lorde argued that reclaiming the erotic disrupts systems that seek to devalue, silence, and disembody Black women. In the South African context, Gqola (5) similarly reminded us that Black women's bodies have long been subject to regimes of control and fear. Her concept of the “Female Fear Factory” exposes how the systemic regulation of women's bodily autonomy reproduces vulnerability and restricts access to pleasure, safety, and agency. Within this context, pleasure becomes a radical and reparative force: a mode of resistance and reimagination that centres Black women’s agency. In spaces like the huis-hospitaal, this pleasure is not reducible to physical sensation; it emerges through ritual, respect, autonomy, and the restoration of dignity in the birthing process.

The paper unfolds in four parts. First, it historicises obstetric violence as a racialised and gendered tool of colonial control, tracing the erasure of Black midwifery in the Cape Colony and Antebellum America. Second, it comments on the marginalisation and significance of TBAs and Black autonomous midwives in democratic South Africa. Third, it presents the huis-hospitaal and the lives of Aunty Faeeza, Aunt Rose, and their grandmother, Ouma, as a counter-archive of care. Finally, within this section, it theorises radical care and pleasure as political and epistemological practices that reclaim reproductive space and knowledge.

## Part I: obstetric violence and the history of midwifery

Globally, the history of obstetric violence is complex and deeply intertwined with racialised and gendered forms of control, which can be seen through the central figure of the Black autonomous midwife. Craven and Glatzel (6) discussed the racial politics of 20th-century public health initiatives that sought to eliminate poor Black midwives through licensing, regulation, and supervision. These ploys reflect a broader colonial project of using medicine as a tool for controlling the reproductive bodies of Black women. This control manifested not only through surveillance and forced institutional births but also via the criminalisation of midwifery outside state-sanctioned spaces, the marginalisation of traditional birthing practices, and restrictions on where and how Black women could give birth.

Obstetric violence, as used here, refers to structural and institutional practices—coercive interventions, neglect, and non-consensual treatment—rooted in a long legacy of regulation and erasure. It is not merely individual misconduct but rather a historically rooted system of reproductive governance. At the turn of the 20th century, efforts to improve maternal and child health care practices came under public scrutiny. As preferences for medicalised hospital births over traditional midwifery grew, so did the institutionalised defamation of Black midwives (6–8). Fraser (9) noted that laws, regulations, and supervisory measures during the 20th century cared more about bolstering a specific racial, gendered social order within the medical realm than maternal and child health. Black midwives were generally perceived as “filthy”, “unhygienic”, “superstitious”, and both racially and professionally inferior to white male doctors (6). Licensing and regulatory campaigns conspired with local law to enforce new training and registration requirements on previously autonomous midwives, specifically aimed at suppressing poor Black midwives. In this way, professionalisation functioned less as a medical advancement more as a disciplinary tool that racialised and delegitimised Indigenous knowledge systems.

Bonaparte (10) noted that this era of professionalisation—through the establishment of an institutionalised, patriarchal medical hierarchy—represented Black autonomous midwives as the Other to the normative, predominantly white, male-led sector of obstetrics and midwifery. This process bolstered the emergence of a white, middle-class cohort of midwives who remained subordinate to and discriminated against by white male physicians. She concluded that physicians' advocacy for regulation was underpinned by a desire to uphold “their medical authoritative knowledge and simultaneously discrediting granny midwifery” (10), and it actively weeded out Black autonomous midwives.

On the Cape frontier, Deacon (1) provided an initial understanding of the history of midwifery in South Africa, which, much like its American counterpart, is entangled in similar kinds of racialised and gendered histories. She began by stating that the paucity of research on this matter stems from the relative absence of the 19th-century Black autonomous, traditional midwife in public archives, and her argument focused mainly on accoucheurs and their relationships with midwives (1). Deacon identified the 19th century as a pivotal moment, marking a shift not only in birthing practices but also the consolidation of patriarchal medical authority, as men increasingly claimed power over women's reproductive bodies through gynaecology and obstetrics. This period saw the rise of male midwives or accoucheurs (1). Like in the United States, training, licensing, and supervision programmes for midwives were implemented to suppress traditional, Indigenous healing practices among Black midwives. Khoisan midwives were perceived as immoral, primitive, dirty, uncivilised, and superstitious “pretenders of great skill in herbs and plants” (1, 11). Deacon noted that “Khoisan women bore the brunt of discrimination and racism and were blamed for the source of diseases because of their filthy and unhygienic lifestyles” (1). These racialised assumptions underpinned formal laws and medical regulation, directly targeting traditional midwifery as a public health risk rather than recognising it as a body of knowledge and care. Not only would this disrupt the flow of traditional knowledge, but it would also shift the sphere of care and knowledge to white male-centred practices.

Taken together, these historical accounts show that the erasure of Black midwives was not a passive process but a violently orchestrated one, enacted through institutional policies, racial ideologies, and gendered medicalisation. Obstetric violence, then, is not an aberration but a structural feature of the colonial medical order. But this structural marginalisation of Black midwives did not begin in the 20th century. Its roots extend further back in South African colonial history, where the foundations of nursing and midwifery were already being shaped by racial and gendered exclusions. One of the few historical accounts that traces this genealogy is Charlotte Searle's *The History of the Development of Nursing in South Africa, 1652–1960* (1965), which offers insight into how midwifery was imagined and recorded during the early Cape colonial period.

### Cape midwifery between the 15th and 19th centuries

“Who did the delivery of the first white South African?… Vrouw de Jager being a married woman and more experienced in such matters probably did the delivery, though she probably had the assistance of the two young women” (12).

The erasure of Black autonomous midwives is evident in earlier historical accounts in the Cape, as shown in the work of Searle's (12) *The History of the Development of Nursing in South Africa, 1652–1960*—one of the few pieces of literature that sought to trace the development of nursing and midwifery in South Africa. Her genealogy of midwifery is among the oldest and most revealing examples of the erasure of Black autonomous midwives from the colonial archive. She credits the first delivery and act of midwifery to Vrouw de Jager, who, based on her work, is white and Dutch. However, she proceeded to note that Jan Van Riebeek's 1654 journal reads: *Yesterday, a Hottentot woman was delivered of a child close to the fort, on the bank of the river, beneath the branches which were piled, without assistance from man or woman, just as if she were an irrational animal* (12). This juxtaposition of events frames Searle's genealogy of midwifery in South Africa. The concern with Searle's work lies not only in what she says, but how she says it.

In the early years of settlement, nursing at the Cape revolved around women and childbirth. The Dutch East India Company appears to have adopted a policy of appointing official midwives to its trading stations, with regulations drafted to govern appointment and control of midwives (12). Searle noted how “the principles which they embodied are as valid today as they were three centuries ago… Modern maternal and childcare requires that the midwife should be certificated and licensed and that a doctor, with his superior knowledge of midwifery matters should be associated with the preservation of the health of the nation” (12). Despite these acclaimed “enlightened” regulations, sworn midwives were not appointed at the Cape for many years, therefore leaving women to fend for themselves and each other.

On the one hand, Searle argued that their spirit of “self-reliance” was crucial to the settlement and survival of the new burgers.^1^ Their medical practices relied on imported French Huguenot knowledge, skills, and medicines. These practices were kept alive by the women of their households who were well-versed in the family remedies and medicines of home nursing (12). Key to this practice was their “Huis Apotheek”, a household medicine box that was generally stocked, managed, and safeguarded by elderly women. Searle noted that this “box” was especially important in the hinterlands, where Company midwives could not reach (12). The limited number of company midwives, appointed much later (1807), could not provide for all who required birth attendants and midwives. As the boundaries of settlement extended, more women lived in secluded villages. Thus, the figure of the “ou-tante” was important in these villages (12).

Searle's neatly crafted story of the downtrodden yet resilient and skilled settlers elevates the “ou-tante” above the elderly slave women who “occasionally acquired skills” in midwifery and acted as a midwife to their owner's wife and other enslaved women (12). This rhetorical move reinforces a colonial narrative that naturalises white women as carers and moral actors while positioning Black enslaved women as peripheral, accidental practitioners.

Searle recounts that Hon. Pieter van Helsdingen took his slave, Claasje, to Batavia to attend to his wife during the trip. Slave owners were responsible for the costs of transit as well as a security deposit in case the enslaved person died (12). Furthermore, she mentioned that in the 16th century, the first known private nurses were enslaved people. With the rising deaths of colonist physicians and a severe shortage of Company-appointed midwives, enslaved survivors of the smallpox epidemic were used as nurses to assist the sick (12). She also wrote at length about the named, official midwives who did not come from the Netherlands but were longer-settled, white elderly women who were certified and paid as midwives by the Company. Among these women, she named Wilhelmina van Zijl (1751), Agetha Bloom (1763), and Catherine Visagie, who were qualified midwives “drawn from a stable social class”, praised for their professionalism, decorum, and behaviour (12). Searle did not name the enslaved people whose contributions to midwifery were equally significant and valid. Her account thus reproduces the racial hierarchy of the colonial state within the very structure of historical narration. By naming white midwives while omitting the identities and knowledge of Black enslaved midwives, the archive constructs a genealogy of care that is exclusively white and professionalised.

Deacon (1) acknowledged the cultural and proximate closeness of enslaved midwives to the white home, whereas Searle erased it entirely. Searle gave credence to struggling white settlers who sourced their own medicine and kept their newborns and pregnant women alive. While she noted the significance of enslaved people to settlers' survival, those who practiced midwifery remained nameless, and their resourcefulness was not valued. This colonial discourse frames her entire book. Searle presented the reader with named white heroines, who appear in the archive, but left enslaved midwives who were key to settler women's survival before, during, and after birth—nameless and voiceless. These enslaved women have no place in the colonial archive other than behind and between its pages. As Deacon (1) noted, Black autonomous midwives effectively “disappeared” from the public archive. This disappearance was not incidental but the product of colonial documentation practices that privileged biomedical authority while rendering Indigenous knowledge forms illegible. This is not merely an omission. It is a form of structural erasure, embedded in the logics of who is considered knowable, visible, and medically legitimate.

To understand this erasure, we must examine how colonial certification emerged as a racialised technology.

### Growing professionalisation: the school of midwifery in the 20th century Cape

In 1807, the Supreme Medical Committee was constituted to oversee the examination, qualification, and licensure of midwives in the Cape. A prominent figure who headed the “development” of the profession was Johann Wehr, a medical official and accoucheur. He was concerned with the “inadequate and often dangerous midwifery which was practised by unlicensed persons in the Cape” (12). In July 1808, he wrote to the Governor of the Cape, claiming that it would serve the administration if he were appointed as Colonial Accoucheur and thus authorised to train midwives for certification:

“… these several years, there has existed a great want of proper and able midwives in this Colony, and that at present there is an aged woman only excepted, not one midwife professionally or legally, instructed and sworn, only Hottentot woman, Free women of colour and even Slaves, presuming to act as midwives, therefore practise freely and the consequence that must arise therefrom both for mothers and children are obvious… THAT convinced by many proofs of your Excellency's philanthropy and paternal care for the welfare of the Colony, your memorialist further begs leave to pray, that it may graciously please your Excellency to appoint him as Colonial Accoucheur and thus to authorize him to instruct an adequate number of midwives for the town and each district—whence of course would flow the duty for him to assist gratis all the wives and the slaves of poor Inhabitants, in cases where knowledge and strength of a midwife are insufficient” (12)

Johann Wehr's self-assurance was affirmed when the Supreme Medical Committee recommended this appointment as “Colonial Instructor of Midwifery”. In 1810, his preparation for the training of midwives commenced (12). Wehr submitted regulations to the committee: the place of instruction would be his private residence (11 Kasteel Street); the Slave Lodge would serve as the Practical School; no person would be considered a midwife unless [she] attended all three courses of lectures and was personally deemed qualified by Dr. Wehr; and the total number of women to be instructed would be limited to six: four white Christian women and two Malay women (12). The first cohort to be qualified included five white women and two Malay or Free Black women. While non-white midwives were allowed to train alongside white midwives, once certified, they were only allowed to treat non-white people (12).

Before certification, midwives were required to take an oath of office against Dr. Wehr's code of ethics. This oath sought to mould them into respectable and dependable assistants to the doctor' ensuring they would not stray too far out of their line of duty. A midwife was expected to “Obtain immediate assistance from a doctor or an accoucheur when such assistance was indicated… Ensure that she was not too venturesome on the one hand and too difficult on the other and had to guard against becoming confused” (12). For Searle, these expectations held as true in the mid-1960s, when she was writing, as they were in the early 1800s (12).

Despite Wehr's regulations for the Cape, untrained midwives continued to practice in the hinterlands. Searle noted that women were forced to fall back on “ou-tantes”, monthly nurses, and “Hottentot women who had gained some measure of midwifery experience by serving their own tribal women and white women”, while “ou-tantes and monthly nurses acquired considerable diagnostic skill and midwifery experience as the years of their practiced lengthened. This specialised knowledge and the traditions of their art were handed down from mother to daughter or daughter in-law, for the knowledge was not to die with the midwife” (12). For Searle, “what the ou-tantes and monthly nurses lacked in technical and scientific skill they made up in humanity, in empathy and in the observation of their unwritten code”, whereas Hottentot women were in her view, “ignorant, depraved, and dirty” (12). These colonial narratives frame midwifery for the foreseeable future. Deacon's key moment—1865—marks the establishment of a midwifery register as midwives became more regarded in professional circles. Only certified midwives were listed here. Registered midwives would be struck off the register if they violated the code of ethics. No Black autonomous midwife appeared on the list, and this is perhaps where Deacon drew her argument from.

This section shows how colonial medicine crafted a selective historical memory in which only certified white midwives were recorded and valorised, while Black autonomous midwives were rendered invisible. The epistemic violence of this omission is part of the broader story of obstetric control and the racialised structuring of reproductive knowledge that the rest of this paper interrogates.

### Twentieth-century midwifery in the transvaal

From the early 20th century, the state attempted to tighten its control over women's health. This period was saturated with internal debates that simultaneously consolidate and present deep cleavages between different levels of the state and organisations regarding women's health. The following archival research was drawn extensively from the Rand Daily Mail archives, the South African Institute of Race Relations archives, found in the Wits Historical Papers Society, the Intermediary Archive Depot, with specific reference to the Public Health Department and West Rand Administration Board, and the Central Archives Depot in Pretoria.

Burns (13) argued that the pinnacle of midwifery is characterised by the establishment of the Bridgman Memorial Hospital, a site of cross-cultural knowledge and practice. From its inception in 1928, the Bridgman Memorial Hospital envisioned an “ambitious program of midwifery training”, which served as a blueprint for programmes launched in the forthcoming decades (13). The hospital was located between Sophiatown and Vrededorp to address the maternal health needs of Black women (13). Before its establishment, no maternal hospital served Black women. However, this did not mean that Black autonomous midwives could freely practise. Instead, they were increasingly pushed out of public visibility and stripped of authority, as their work was delegitimised by new institutional frameworks demanding certification and conformity to biomedical and Christian ideals. The hospital served as a locus for “civilising” both midwife and patient into the ideals of productive, clean, Christian motherhood (13).

The establishment of the Bridgman Memorial Hospital was a material manifestation of debates and anxieties about race, sanitation, the “black peril”, and the “betterment of poor whites” (13). These anxieties were expressed in the white supremacist state's obsessions with the sexualised and immoral dangers of Black women who began moving into the city and surrounding areas at the time. Swanson wrote, “Medical officials and other public authorities in South Africa at the turn of the century were imbued with the imagery of infectious disease as a social metaphor… this metaphor interacted with British and South African racial attitudes to influence the policies and shape the institutions of segregation… urban public health administration was of considerable importance in accounting for the ‘racial ecology’ of South Africa…” (14). Swanson elaborated on the “interconnections between local state health formation, racial segregation and the representational power of disease and contagion”, which he aptly named the “sanitation syndrome” (14, 15). This metaphor of disease justified both physical and epistemological interventions into Black women's reproductive lives. A piecemeal public health system was built upon the “sanitation syndrome”, which had its roots in 19th-century health initiatives.

Alongside continued racialised concerns about contagion, public health attention shifted toward Black maternal and child health, owing to the new configuration of the city. This shift manifested in a focus on hygiene, health, and motherhood. These discourses framed Black maternal bodies as inherently risky and in need of state regulation while simultaneously undermining the legitimacy of Black midwives as trusted caregivers. Race, gender, and urbanisation were conveyed to press officials to link Black and white maternal and child mortality and morbidity (13). This extended to a concern for Black reproductive bodies and, consequently, for midwives.

As found in the UP digital repository, the 20th century saw the Transvaal passing an Ordinance modelled on its Cape counterpart, which regulated the work of midwives and nurses and established a midwifery register. Charlotte Searle noted that the 1891 Medical and Pharmacy Act made provisions for the regulation of midwives and nurses trained at the school established by Wehr (12). The Transvaal Ordinance originally stated that unregistered midwives who had been practising for at least a year prior to its passing, in theory, could continue practising subject to licensure and registration (16). However, with the state in a flurry over infant and maternal mortality and morbidity rates, at a more central and organisational level, the Transvaal Medical Council opposed the promulgation of new measures to register and investigate midwifery practice (13). This pushback reflects a broader reluctance to recognise alternative models of care, especially those rooted in community-based or Indigenous practices.

### Transition to the Bridgman

The racialised anxieties around public health, specifically related to Black maternal and child health, saw the implementation of various policies and institutions aimed at controlling the reproductive bodies of Black women and the midwives who tended to them. One such institution was the Bridgman Memorial Hospital. The hospital was designed to regulate and manage Black reproductive health. It was a site of both inclusion and assimilation: young Black women were trained as midwives but within a narrow model that demanded compliance, discipline, and Christian moral order. Bridgman's founders dreamed of establishing a space governed by Christian values. They sought to mould young women into good mothers and midwives. They called for sobriety, dignity, adherence to Christian values, and the scientific management of the body (13).

Following the pre-1928 anxieties over racial contamination, the establishment of the Bridgman Memorial Hospital sought to erect strict boundaries between the economic and social lives of Black and white women, motivating the establishment of a maternal welfare institution that catered for Black women specifically (13). Clara Bridgman and Edith Reinhalt-Jones were important figures in its establishment (13). Despite its constricting and somewhat paternalistic ideology, the Bridgman prided itself as a training space for Black midwives and nurses. However, this training did not confer full authority or autonomy—trained midwives often remained subordinate within hierarchical hospital settings and could rarely open independent practices. The institution accepted young Black, unmarried women (13)*.* The Bridgman served as the first and, for a long time, the only site of large-scale midwifery training for Black women.

Rather than marking a rupture from colonial practices, the Bridgman Memorial Hospital extended its logic: regulating who could birth, who could assist, and under which values. The shift from autonomous community care to regulated institutional care was justified under the guise of public health, but in effect, it dispossessed Black midwives of their knowledge and communities of their trusted caretakers. In its institutionalisation of midwifery, the Bridgman served as a locus for “civilising” both midwife and patient into the ideals of productive, clean, Christian motherhood.^2^

However, as forced removals reached violent heights in the 1960s and grand apartheid enforced harsher and harsher segregationist ideals—with Sophiatown at the heart of it in Johannesburg—the Bridgman, seen as a space particularly threatening to said segregationist ideals because it positioned Black women in close proximity to white neighbourhoods, was forcibly closed in 1965. Black mothers in Johannesburg were left with Baragwanath Hospital as their only maternal care facility, and midwifery training was absorbed into nursing training. This shift did not just close a building. It marked the end of a chapter on Black midwifery autonomy. By folding midwifery into nursing, the state subordinated reproductive care to a broader medical bureaucracy, effectively erasing the role of the midwife as a specialist, community-embedded figure. The consequences of this erasure still echo in the marginalisation of midwifery today.

## Part II: where are the midwives now?

It has been a pleasure to return to the research I began during my MA,^3^ when my questions were urgent but my methods less refined. I drew on Deacon's (1) argument that the Black autonomous midwife disappears from the public archive not because she ceased to exist but because the archive itself was structured to forget her. In response, I argued that these women had not vanished entirely but lingered quietly, like the faint imprint of a lost paperclip.

Looking back now, my re-reading feels necessary. I was less attuned to how autonomous midwifery has always exceeded the limits of the public archive. Writing this paper has allowed me to shift my focus and rethink my initial outcomes. I am no longer just trying to make the midwife visible. I am asking how Black women's care practices have lived beyond, around, and in spite of the systems that sought to discipline them.

Burns' (13) work has been central to this shift. Her analysis of the Bridgman Memorial Maternity Hospital remains one of the most critical accounts of medical modernity in South Africa. She wrote that the Bridgman was not only a hospital, but a “massive project to train and certify a cadre of black midwives and as a space for scientific research concerning their bodies, birthing capacities, and the gynaecology of black women” (13). For Burns, Bridgman was a site where colonial and Christian ideals of respectability and motherhood were mapped onto reproductive care, where midwives were brought under surveillance, and where obstetric authority was consolidated through the figure of the trained nurse (13).

However, Burns (13) read the archive carefully, showing how Black women continued to do the work of care within this tightly governed space. She introduced us to figures like Louis Mvemve [see (13), Chapter], the midwives of the Alexandra Clinic (13), and women like Rosina Kotane (13), who sought medically assisted births while remaining embedded within community networks. These women, Burns reminds us, undermine the idea that institutions like Bridgman operated as totalising systems. “Three generations of Bridgman midwives could meet with one another” (13), she writes, because their work did not begin or end at the hospital gates. Their knowledge travelled. Their care persisted.

During my MA, I argued that while South Africa's transition to democracy ushered in meaningful reforms, the ideologies of colonial and apartheid reproductive governance were never fully dislodged. Instead, they were repackaged as progressive public health measures, often through international development discourses such as the WHO's Safe Motherhood initiative. This initiative framed maternal mortality as a violation of women's rights and urged governments to allocate political and financial resources to address it (18). However, these efforts often re-inscribed a racialised logic of control. The Black woman body, now framed as the site of humanitarian intervention, remained subject to surveillance, regulation, and biopolitical management.

Nowhere was this more visible than in the post-apartheid state's approach to traditional birth attendants (TBAs). Rather than being recognised as autonomous practitioners rooted in community-based knowledge systems, TBAs were positioned as risks to be mitigated. Their roles were reduced to that of informal extensions of the state's biomedical infrastructure—useful for outreach, referrals, and compliance but not for defining or shaping care itself. These dynamics were intensified during the HIV/AIDS crisis, where public health discourse became highly centralised and risk-averse. TBAs were framed as vectors of infection rather than as partners in care (19).

One of the clearest examples of this co-option can be seen in Peltzer and Henda's (19) study, which piloted a 4-day training programme for 50 TBAs from two clinics in the Eastern Cape. The programme aimed to improve antenatal and postnatal care, promote HIV prevention, and shift TBAs' practices to align with biomedical protocols (19). Although the study reported increased clinic referrals and improved knowledge about nevirapine administration, it was premised on the assumption that TBAs were unhygienic, underqualified, and in need of correction (19). Traditional practices were acknowledged but never engaged on their terms. The training programme operated not as a dialogue but as a disciplinary intervention, offering TBAs a narrow script for referrals and compliance while demanding nothing of the healthcare system in return.

This framing was also institutionalised at the policy level (20). The Policy on Traditional Health Practitioners placed TBAs under a logic of registration and compliance. State recognition was conditional: TBAs were required to conform to biomedical norms, and their practices were regulated rather than affirmed (21). Rather than transforming the structural inequalities that shaped Black women's birthing experiences, the state redirected responsibility onto TBAs, further entrenching the historical pattern of using Black midwives as expendable adjuncts to state health systems. This echoes findings from Abrahams et al. (22), who showed how midwives, although often central to women's care-seeking, were undermined by broader systemic failures.

Looking back, I now recognise that my initial reading treated the traditional birth attendant primarily as a passive subject of state intervention: an object of regulation and training. While this framing was analytically valuable for identifying the continuities between colonial, apartheid, and post-apartheid reproductive governance, it risked flattening the complexity of TBA practices and misrecognising their agency. More recent scholarship and emerging empirical work paint a more layered picture. Rather than disappearing into the machinery of the state, TBAs continue to act as vital intermediaries, caregivers, and knowledge-holders in their own right.

Garces et al. (23) revealed that TBAs remain active across many parts of the Global South, particularly in rural and marginalised areas. Despite a global policy shift toward facility-based births, many women continue to seek out TBAs not solely out of necessity but because they are trusted, known, and embedded in the communities they serve. As the authors note, “TBAs often serve as a bridge between the community and the formal health system” and play crucial roles “including support and advice to women during pregnancy and childbirth” (23). These practitioners straddle multiple roles: “cooking or caring for other children around childbirth”, acting as herbalists, and drawing on “experience acquired through apprenticeship or self-teaching” (23). Far from being obsolete, TBAs remain “a crucial part of health care for women living in rural and underserved areas”, especially where formal services are inaccessible or alienating (23).

In the South African context, Musie and Mulaudzi (24) complicated the assumption that TBAs are uniformly excluded or delegitimised. Their study found that “only 30.8% of midwives knew of the roles of traditional birth attendants for maternal and neonatal healthcare” and that many midwives held “negative attitudes towards collaborating with TBAs during intrapartum and postpartum care” (24). However, the study also found that 70.4% of midwives supported collaboration with TBAs for roles such as “accompanying women to health facilities”, especially in antenatal contexts (24). These findings reveal the institutional ambivalence toward TBAs and the possibilities of limited, meaningful partnerships rooted in community trust.

Similarly, Ngunyulu et al. (25) foreground the enduring role of TBAs in postnatal care in Limpopo Province. In their study, TBAs described rituals such as keeping mother and baby in isolation for 6 weeks “to exclude evil spirits (prevent infections)”, preparing “special warm food to maintain good nutrition”, and ensuring that women “delay sexual resumption to allow recovery of reproductive organs” (25). These practices are not marginal add-ons to clinical care but constitute a parallel system of healing rooted in indigenous cosmologies and embodied knowledges. As the authors note, TBAs “play a crucial role in the care of women during pregnancy, birth and puerperium within communities” (25).

What these studies illuminate is that TBAs are not relics of a pre-modern past, nor are they simply passive tools of the state. They occupy a contested and creative space within the reproductive landscape where they are negotiating regulation, navigating ambivalence, and continuing to prioritise care on their own terms. As Hastings-Tolsma et al. (26) reminded us, birth remains a site of deep vulnerability. Their qualitative study across South African birth settings noted that women often feel “scared and uncertain”, “unwanted”, and experience care as “non-caring and lacking in compassion” (26). Yet in midwife-led or home-based care, women described entering what the authors called a “cocoon of compassionate care”: a space defined by presence, respect, and warmth (26). These cocoons are sites not only of survival and safety but also of possibility where care becomes life-affirming and where the seeds of pleasure-centred birthing practice take root.

This is precisely what the huis-hospitaal in Eldorado Park embodies. In the care work of the voetvroue, the home becomes more than a refuge. It is a site of sacred, relational, and pleasure-affirming birth practice, where Black women reclaim control over their reproductive lives and forge intimate, life-giving commons of care.

## Part III: towards a living archive: voetvroue and the huis-hospitaal

Aunty Faeeza described their home in Kliptown as barely big enough for their own family to live in, let alone to function as a *huis-hospitaal*. This was her everyday, where domestic life and reproductive labour constantly intersected. She was constantly encountering pregnant women, who would stay for weeks on end until their babies were born. They occupied most of the space in the house, leaving Faeeza and Aunt Rose to use the pantry as a place to lay their heads.

Catching babies was a norm in the lives of Aunty Faeeza and Aunt Rose, so much so that they took it on once Ouma deemed them fit enough. For them, birth and delivery happened in the *huis-hospitaal*—a centre of care that women from the community would be “booked” into until they gave birth. No general timeline was known, so they operated on the premise that birth would occur at any moment. Because the space was so populated, women were forced to give birth in the very spot where they had spent weeks. If they received the bed, they delivered it there instead. However, if all they could receive was a mattress on the floor, they delivered it there instead. It worked on a first-come, first-serve basis, but they were best catered for. Every room was occupied by mothers from the community at any given point.

This spatial logic, where scarcity and care were negotiated daily, illustrates the huis-hospitaal as a radical re-imagining of institutional birthing: one rooted in improvisation, reciprocity, and affect.

Yet alongside hardship and urgency, there was also pleasure—what Lorde (4) framed as the erotic: a deeply felt sense of aliveness, connection, and purpose. Aunty Faeeza laughed when recalling how she and her siblings trampled laundry in buckets with their feet while their grandmother, Ouma, hummed softly nearby. These were not just tasks—they were gestures of intimacy, rhythm, and joy. There was pride in making the huis-hospitaal function, in wrapping babies, preparing coffee, and witnessing new life. Amid the precarities of informal healthcare, the act of care itself became a site of pleasure, of knowing that one's hands could hold, soothe, and sustain.

Aunty Faeeza spoke in a tone of frustration as she described how, even though they lived in a two-bedroom backroom, every room was occupied, so much so that Aunty Faeeza had to sleep in the pantry cupboard. Her job, as one of the grandchildren, was to collect sheets and help wash them. We shared in fond memories of our grannies making our little feet tramp on laundry in a wash bucket to help clean it. She loved her childhood, but a large part of it was spent tending to the huis-hospitaal. Similarly, Aunt Rose, who was older then, started assisting in deliveries from the age of 12. She, her sisters, and her brothers were all put to work to ensure that women had the care they needed. Interestingly, she also described how her brother learned to catch babies. By the time she was 19, Ouma often let her catch babies alone. An essential but dangerous practice that saw both her and Ouma in precarious situations. She mentioned a time when threats were brought upon their lives because a woman had asked for an emergency birth and they allowed her in, only to find out that she had delivered a stillborn in her water bath while they were prepping her for delivery. This angered the woman's family to the point where they issued threats of violence against Aunt Rose and her Ouma.

During delivery, Faeeza and Aunty Rose note how they did not administer any pain medication in fear of the side effects it may have, especially considering that, most of the time, they did not know the history of her patient and the condition of the foetus. What was instead administered was strong, black coffee. This improvisational pharmacology, grounded in intergenerational and spiritual knowledge, reflects a different epistemic framework, one that prioritises embodied responsiveness over medical protocol. Birthing was not done in any particular position for any specific reason. Instead, it depended on the space available.

True to Davis-Floyd et al. (27), it is inaccurate to view “lay” midwifery as biomedicine's “evil twin” and simultaneously inaccurate and demeaning to romanticise it as a realm where no wrong occurs. The process of delivery was dangerous and volatile:

Aunty Rose: Nee, dit was hard my kind. First of all, we only knew how to deliver babies because my mother was mos qualified. Jy moet die kop hou, draai die kind om, maak laat die skouer uitkom, dan ruk jy. And unfortunately, not all babies survived. Een minute huil die kind en die next is die kind lui. Dit was nie easy nie en die huis was altyd vol van swanger ma'ens. Kyk, daar was een aand, mama ‘n baby gaan vang by Mr Hajee, daar agter onse straat gewees en dit was net ek en my groot broer by die huis. Toe's daar ‘n vrou ook by die huis wat ‘n baba kry. Aunt Mama was haar naam. Ons try om die baby te kry. Ons weet mos wat om te doen, maar ons kan nou nie hier kom om die slym uit die mond uit tehaal nie. Ons haardloop. Toe my ma kom het sy net so gemaak, toe haal sy die slym uit. We saw all kinds of things man. Hou noem jy dit? We called it a jelly baby. Dan moet jy die kind doktor toe vat om die skin te prick sodat die water kan uitkom. Ander tyds kry jy vrouens wat druk en druk maar niks kom uit. Jy sê mos ‘n phantom pregnancy. Ons het als by die huis gesien man.^4^ (1 September 2020)

Aunt Rose describes how they had a standard process of delivery because of her mother's training as a midwife. They would ensure that the head of the baby was firmly held. They then turned the baby around and gently pried the shoulder out. The rest of the body would follow. Her experiences of delivering babies were filled with varying kinds of scenes, from stillborns to “jelly babies” to babies born with *hydrops fetalis*. She particularly details how delivery went either way for the mother and the child. Faeeza described it as having one foot on the earth and another in the grave, and this was an everyday experience for Faeeza and Aunt Rose. The *huis-hospitaal* housed varying experiences of birth. These stories narrated by Aunty Faeeza and Aunt Rose are raw, real, and ghastly and reveal the house not just as a site of refuge but one of risk, improvisation, and extraordinary skill.

Importantly, it was a non-monetary practice. Women who sought care could do so without incurring costs. This applied to time spent before, during, and at least 7 days after delivery. All Aunt Rose, Aunty Faeeza, and their Ouma asked for were gifts or referrals to “keep their paths open”. This gift economy reflects a radically different ethic of care, one structured by reciprocity, spiritual obligation, and communal accountability. The huis-hospitaal is a space of refuge for women in the context of limited access to public maternity healthcare facilities and for voetvroue who were actively sidelined by the biomedical sphere. Midwifery regulation and control have historically run along the explicit lines of race, gender, and class, morphing those deemed acceptable into a palatable class of subordinate birthing assistants and rooting those deemed the very opposite out. However, in the very midst of this history, Black autonomous midwives have continuously carved out a place for themselves and for the women they serve. The huis-hospitaal is not only a physical structure. It is a fugitive infrastructure of care, emerging from the cracks of exclusion, carrying forward a spiritual and political legacy of resistance.

Despite South Africa's transition to democracy, little has changed in terms of funding and accessibility. Eldorado Park and its surroundings remain underfunded and subsequently crippled by drugs, poverty, and unemployment (28). The commons made by the midwives of Eldorado Park provide women with a non-monetary space to receive holistic care. These spaces were in formation before Eldorado Park's inception, providing, as Harney and Moten (29) argued, spaces of refuge that enable the birth of commons. This space not only provided refuge but also fostered new forms of care and belonging, rooted in histories of dispossession and marginalisation (30).

In doing so, it stood as a counter to the oppressive colonial and apartheid medical regimes. These women carved out spaces of care and refuge in the bowels of power. Through their dynamic, communal birthing practices, they constructed alternative forms of care and survival, challenging the racialised and gendered control imposed by these biomedical regimes. These practices resist the kind of institutional sanitisation that Fraser (31) critiqued—where discomfort and complexity are often flattened to present neat, authoritative versions of history. These practices not only offered refuge from biomedical violence but also created space for joy, dignity, and a deeply felt erotic knowledge, where Black women reclaimed pleasure in their roles as carers and creators of life. The huis-hospitaal, far from being a leftover or marginal space, became a radical “commons of care”—a refuge where women could access non-monetary care that was culturally resonant, asserting their agency over their bodies in the face of systemic neglect. They had a choice over how their baby was delivered, in which position or setting they wanted to give birth in, and how long they wanted to stay at the huis-hospitaal and were also cared for up to 10 days after birth.

The history of the huis-hospitaal and the work of voetvroue illuminate the broader argument of this paper: while colonial and apartheid medical regimes sought to regulate and control the reproductive health of Black women, their reach was not totalising in erasing the autonomy of Black women. Voetvroue, operating in the interstices of power, created counter-spaces to the racialised hierarchies of biomedical care. In doing so, they act as subversive spaces to the racial and gendered violence and disciplinary nature of state healthcare, providing a model for community-centred, non-monetary healthcare that continues to resonate in post-apartheid South Africa. In reclaiming the right to give and receive care outside of state control, the voetvroue of Eldorado Park make visible a form of reproductive justice rooted in intimacy, memory, and collective strength. Their practices remind us that resistance is not only a refusal of violence but also an embrace of pleasure, dignity, and life-making.

## Conclusion

Despite the transition to democracy, the legacies of obstetric colonial and apartheid violence over Black women's bodies still exist. Underfunded and underserved communities like Eldorado Park still suffer under systemic neglect, echoing the very inequalities that these “commons of care” were formed as a result of. However, these commons of care have carved out a space where agency is prioritised. The huis-hospitaal serves as a physical manifestation of this and exemplifies how Black women continue to resist the ongoing racialised control over their reproductive health, offering a vision of resilience and hope in the face of historical and ongoing forms of gross exclusion, abandonment, and violence.

The practices of voetvroue do not merely resist. They remake. They create generative spaces that honour birth as a site of dignity, relation, and sacred knowledge. In doing so, they challenge the supremacy of biomedical models not through formal opposition but through everyday practices of care that centre pleasure, autonomy, and cultural knowledge.

The huis-hospitaal and the work of voetvroue exemplify how pleasure, conceived relationally and politically, can be a reparative force in contexts of systemic harm. It allows us to re-imagine reproductive justice not only as survival but also as flourishing.

Their practices are disruptive to dominant biomedical discourse, even though they do not intend to be—their contributions need not be acknowledged by any hegemonic power for it to be as effective and apt as it is. They reveal the enduring power of community-based care that permits reclaiming the emotional, physiological and even spiritual dimensions of birth and reproduction, recognising it as an embodied, empowering experience. By centring pleasure, they have opened up new ways of seeing reproductive health: ways that have honoured the complexity, agency, and dignity of women amidst historical violence and present-day structures that continue to discipline and devalue Black reproductive life. These women created not only refuge but also moments of dignity and joy, where their hands, rituals, and knowledge reasserted Black women's right to experience care not as charity but as love, connection, and purpose. Ouma has long passed on and the huis-hospitaal was razed by the apartheid government in an effort of “slum clearance” in Kliptown during its tenure. Unfortunately, a space that sought to evade the violent machinery of apartheid was eventually violently destroyed by it. Its destruction is part of a longer history in which Black midwifery practices were not only physically dismantled but also systematically erased from the public archive, a disappearance driven by colonial documentation practices that privileged biomedical authority and rendered Indigenous knowledge forms illegible (32). This loss is not only material but also epistemic and spiritual. However, its trace remains, carried in story, memory, and practice. The writing of this paper is an act of honouring that trace. It hopes that the legacy of the huis-hospitaal and all that it embodied is celebrated and remembered. A small two-bedroom backroom, headed by ordinary community women, served as a space that offered a re-imagining of community-based healthcare. It was physically small but epistemologically and historically monumental.

## Footnotes

^1^Afrikaans for “citizens” or settler-citizens.

^2^Despite Bridgman’s commitment to moulding disciplined Christian midwives and mothers, Burns beautifully noted that, “the will and subjectivity of black women at prenatal classes, in examination rooms, and on birthing tables, arrested the full implications of scientific inquiry based on racial taxonomies and interrupted easy dichotomies between notions of civilization and primitivity so present in expert discourse across South Africa in the first half of the century” (13). Hence, Burns’ argument that constructs of “western” and “indigenous”, as we anthropologically know it to be, are not as fixed as they appear to be (13). Women who assisted births at the Bridgman undermined simplistic assertions that discourses of science created totalising institutions or dioramas out of the lives of black women in Johannesburg (13).

^3^Writing this reflection is necessary for several reasons. First, these initial thoughts were previously published in *Where Have the Midwives Gone?* (17), and I feel it is important to outline my process of re-engagement and reflection. Second, researcher reflexivity is not only a methodological necessity but also a decolonial intervention. One that reminds us that it is intellectually and ethically necessary to re-read, re-think, and revise. Reflexivity foregrounds the reality that knowledge-making is ongoing and situated and that returning to our past work can help us produce more just and nuanced scholarship.

^4^The dialogue reads “No, it was hard my child. First of all, we only knew how to deliver babies because my mother was mos qualified. You must hold the head, turn the child over and make the shoulder comes out then you pull. And unfortunately, not all babies survived. One minute the baby is crying and the next it is still. It was not easy and the house was always filled with pregnant mothers. Look, there was one evening when my mother left to catch a baby by Mr Hajee’s daughter behind our street and it was just myself and my brother at home. There was also a woman, her name was Aunt Mama. We tried to catch the baby. We knew what we had to do but we could not get the slime out of the mouth. We ran to my mother and she came back to remove the slime. We saw all kinds of things. What do you call it? A jelly baby. Then you have to take the child to the doctor to prick the skin so that the water can come out. Other times, you get women who push and push but nothing comes out. You call that a phantom pregnancy. We saw everything at that house”.
